# Barriers and Enablers of Diabetes Self-Management Strategies Among Arabic-Speaking Immigrants Living with Type 2 Diabetes in High-Income Western countries- A Systematic Review

**DOI:** 10.1007/s10903-023-01576-0

**Published:** 2024-01-17

**Authors:** Anwar Noor Althubyani, Sabrina Gupta, Clarice Y. Tang, Mehak Batra, Rahul Krishna Puvvada, Peter Higgs, Markandeya Joisa, Jency Thomas

**Affiliations:** 1 Department of Microbiology Anatomy Physiology and Pharmacology (MAPP), School of Agriculture Biomedicine and Environment (SABE), La Trobe University, Melbourne, Australia; 2 Department of Public Health, School of applied science, University of Tabuk, Tabuk, Saudi Arabia; 3 Department of Public Health, School of Psychology and Public Health, La Trobe University, Melbourne, Australia; 4 Department of Physiotherapy, School of Health Sciences, Western Sydney University, Penrith, Australia; 5 Institute of Health and Sport, Victoria University, Melbourne, Australia; 6 Department of Pharmacy Practice, JSS College of Pharmacy, JSS Academy of Higher Education and Research, Mysuru, Karnataka India

**Keywords:** Arabic-speaking background, Self-management, Type 2 diabetes, Barriers, Enablers, Systematic review

## Abstract

The aim of this review is to investigate barriers and enablers of diabetes self-management strategies among migrant Arabic-speaking background [ASB] individuals living with type 2 diabetes in high-income Western countries. Despite living in high-income Western countries, individuals from ASB are perceived to have difficulties adopting self-management strategies and this necessitates gaining an understanding of factors that may impact the uptake of these strategies. Ten studies are included in this review: five quantitative and five qualitative. Quality assessment was conducted using the Joanna Briggs Institute Critical Appraisal and Hawker tools. The findings of the quantitative studies were descriptively analysed, while thematic analysis was performed for the qualitative studies. The results indicate that individuals from ASB are perceived to have low levels of adherence to diabetes self-management. It is also suggested that participants who did not complete high school have poorer glycaemic control compared to those with a high school qualification (30 vs. 16%). Regular exercise was reported to be less likely to be adopted by ASBs homemakers, and those who were unemployed, by 82% and 70%, respectively, compared to those employed (homemakers: OR = 0.187, P = 0.006; 95% CI = 056–0.620), (unemployed OR = 0.30, P = 0.046; 95% CI = 0.093–0.980). Cultural, social, religious beliefs, lack of knowledge and language barriers are some of the factors identified that impact self-management among ASB individuals. It is suggested that diabetes self-management education program (DSME) tailored to ASB immigrants culture may be an effective way to encourage them to uptake self-management strategies.

## Introduction

International migration has grown in the recent years, with the number of persons living outside their country of birth or citizenship reaching 281 million in 2020, up from 221 million in 2010 [1]. In 2020, immigrants comprised nearly 15% of the total population in high-income countries, compared to less than 2% in middle- and low-income countries [1]. Lifestyles for many immigrants may change when adapting to and settling into host countries, and over time this may predispose them to an increased risk of non-communicable diseases (NCDs), such as type 2 diabetes and other chronic diseases [2]. Type 2 diabetes can severely impact an individual’s functional capacity and quality of life, leading to significant morbidity and premature mortality, and is considered a leading cause of death, with approximately 6.7 million deaths globally in 2021 [3]. Type 2 diabetes mellitus (T2DM) is the most common type of diabetes, representing 90% of all diabetes cases [3]. Immigrants from Arabic-speaking backgrounds (ASBs) are one such ethnic group that have higher rates of T2DM in comparison to host populations in high-income countries [4]. For example, in a US study, it was found that Arab Americans were 5–8% more likely to be diagnosed with diabetes compared to the general US population [5].

Global migration patterns and a trend towards multicultural societies suggest that there are increasing numbers of Arabic-speaking immigrants in high-income countries [6]. Given the high rates of T2DM amongst those who are ASBs immigrants, effective self-management techniques must be considered. Self-management, is defined as the day-to-day activities or actions an individual must undertake to control or reduce the impact of disease on their health and wellbeing, as well as to prevent complications [7]. Regular engagement in diabetes self-management (DSM) has been linked to improved health outcomes, such as improved glycaemic control, fewer complications [8], better quality of life [7, 8], and a reduced risk of diabetes-related death [9]. DSM involves activities such as medication adherence, healthy eating, being active, and regular monitoring of blood glucose levels, which are all necessary for the successful management of the disease [10]. It is commonly known that there are a wide range of factors that may lead to the progression of complications related to T2DM. They may include lack of knowledge regarding DSM and communication barriers between health-care providers and patients. Such factors may influence decision making processes for DSM.

Along with adapting to a new country, immigrants face additional challenges in managing chronic diseases such as T2DM due to a lack of culturally apt education programs and of trained health-care providers who have awareness of cultural issues [11]. These have been found to be correlated with poor DSM among immigrants [12]. It has also been shown that geographical distance from the country of origin, cultural differences, limited social networks and lack of social support in the host country may also affect immigrants’ abilities to deal with chronic diseases [13].

Multiple studies have demonstrated that culturally appropriate self-management interventions can have a positive impact on diabetes control and quality of life in immigrant populations [14, 15]. For example, Scardera et al., in a systematic review, found that self-management interventions can be beneficial for immigrant populations, but they need to be culturally adapted to be most effective [16]. Kim et al., reported that a culturally tailored self-management program improved diabetes control and self-confidence among Korean immigrant women [17]. An et al., found that a culturally adapted diabetes self-management program was effective in enhancing diabetes control and health-related quality of life in Chinese immigrants in the US [18].

While there have been multiple studies in different cultural groups supporting the importance of culturally appropriate interventions, there have not been any studies reporting on the impact of culturally appropriate interventions for people with T2DM from ASBs. In order for future research to understand which aspects of culture need to be further explored when designing a culturally appropriate intervention for people from ASB living with T2DM, it is imperative to identify barriers and facilitators of DSM among Arabic-speaking immigrants. To date, there are a few studies [19, 20] which have investigated the factors impacting DSM among such immigrants, yet no systematic review has been undertaken to synthesize these findings. Therefore, in this review we seek to systematically synthesize the evidence on factors that facilitate or impede T2DM self-management among ASB immigrants in high-income Western countries.

## Materials and Methods

This systematic review was conducted based on the PRISMA guidelines. The protocol for this review was registered on PROSPERO (registration number: CRD42020190347).

### Search Strategy

The first search was carried out between April and June 2019, with search terms related to the following: Arabic speaking, Middle East, immigration, migration, refugee, diabetes and non-insulin dependent (Table 1). The search was limited to humans (see 2.2.2, item 3, below) and to the English language. The search was updated between May and June 2022, and one paper was added to the final review [21]. The Boolean Apparatus and MeSH terms* were used. The databases searched included PubMed, CAB Direct, Medline, EBSCO [CINHAL], PSYCINFO, SocINDEX, Sociological abstract, ProQuest central, Web of science, Google Scholar [first 10 pages], Scopus, and Embase. In addition, manual screening of the reference lists of the screened studies was performed. All studies related to T2DM self-management amongst ASB individuals in high-income Western countries were selected and imported to Covidence, a web-based program used to assist with systematic reviews [22]. Two reviewers, AA and RK, independently participated in the selection of the studies. Titles and abstracts were screened by these two reviewers to identify articles that met the inclusion criteria. Any disagreements were resolved by other researchers (SG and CT). Full-text papers that met the inclusion criteria were independently screened by three reviewers (AA, MB and RK) for the data extraction.

**Table 1:** Search terms used for the study

Search terms
Arab* OR “Arabic speaking” OR “Middle east*” OR Gulf
migra* OR immigra* OR refugee* OR vulnerable OR minorit* OR emigra* OR “asylum seek*”
Type 2 Diabetes OR Diabetes Type 2 OR type II diabetes OR Non- insulin Dependent Diabetes OR diabetes Mellitus
1 AND 2 AND 3

### Eligibility Criteria

#### Inclusion

1. Studies (both quantitative and qualitative in design) that included Arabic-speaking immigrants living in high-income Western countries [23].
2. Aged 18 years and above and living with T2DM.
3. Studies that have explored at least one or more DSM strategies.

#### Exclusion

1. Studies that were conducted outside high-income Western countries.
2. Studies related to gestational diabetes or type 1 diabetes.
3. Animal studies.
4. Case studies, case reports, narrative reviews, systematic reviews, meta-analysis, conference abstracts. Clinical trials.
5. Full-text studies that were not in English.

### Data Extraction

Data were extracted independently by AA and RK using a customized form (Tables 2 and 3). Study results and relevant information on DSM were extracted and are presented using the form. SG, JT, CT, and MJ reviewed the data extraction documents.

**Table 2:** Summary Characteristics of qualitative studies

First Author, Pub.Year.	Study Settings/population/duration/sample size	Study’s Aim	Study design/tools used	Findings
Alzubaidi et al., 2015	Many health care settings including diabetes outpatient clinics in two tertiary referral hospitals, six primary care practices and ten community centres.	A comparison between Arabic-speaking and English-speaking patients with type 2 diabetes regarding the experience of using medications and associated issues	Total participants N = 100 Arabic speaking n = 60 English-speaking n = 40 face-to-face semi structured individual interviews and group interviews. The questions were related to medicine taking behaviours and this include: Knowledge, views and communication with health care providers about medicine.	1- Arabic-speaking individuals (ASB) often neglect to take medication due to their skepticism towards Western medicine. Furthermore, they tend to deliberately postpone seeking medical care when signs of diabetes appear. 2- There exists a belief among ASBs that enduring illness brings them closer to God. 3- ASBs expressed dissatisfaction about the lack of sustained dialogue with healthcare professionals. Instead, they lean significantly on relatives and friends for guidance concerning their prescribed therapies. 4- The Arabic-speaking community tends to have a negative perception of diabetes, viewing it as a social stigma. They believe that individuals with diabetes no longer fully integrate within their society.
Bertran et al., 2015	Self-identified Arab or Arab American 18 years and above with diagnosis of diabetes. Communities and pharmacies in Michigan N = 23	Understand the barriers and facilitators of diabetes self-management education (DSME) among Arab Americans.	3 Focus groups sessions used with questions aimed to explore the perceptions on DSM.	Cultural views on gender roles reveal inappropriateness of the female participants participation in mixed-gender exercise programs. Female participants expressed satisfaction with the physician’s non-interactive and directive style of interaction. Participants participation during the Ramadan, a cultural tradition, could supersede the advice of the provider and can impact the DSME The major barriers for DSME are the lack of available educational and support resources. Physician’s interaction style and comprehensiveness of care were the two most important features influencing participants’ views of patient-provider interactions.
Alzubaidi et al., 2015	Recruitment occurred at diabetes outpatient clinics in 2 tertiary referral hospitals, 6 primary care practices and 10 community centres	To explore the decision-making processes and associated barriers and enablers that determine access and use of healthcare services in Arabic-speaking and English-speaking Caucasian patients with diabetes in Australia	Total participants N = 100 Arabic speaking n = 60 English-speaking n = 40 face-to-face semi structured individual interviews and group interviews. Duration of interviews ranged between 30 and 110 min The questions were related to who ASB approached when symptoms of diabetes developed, when and how they made a decision to access and use healthcare services, what are some associated barriers and enablers, perceptions about healthcare professionals, and knowledge of available diabetes healthcare services	1- English-speaking individuals typically consult a general practitioner within days or weeks of noticing symptoms, whereas Arabic-speaking individuals usually delay such visits for months. 2- Compared to their English-speaking counterparts, Arabic-speaking immigrants have demonstrated less awareness of the non-medical services currently accessible for diabetes management. 3- The strong belief held by Arabic-speaking persons, encompassing ideas like “human life is ephemeral” and the notion that seeking medical aid is futile as death is inevitable, impacts their health behaviors. Additionally, they perceive enduring illnesses such as diabetes as a sign of proximity to God (Allah) and a path to absolution of sins. Consequently, their motivation to participate in self-management of diabetes is diminished.
Fitz et al., 2016	Health care facilities in Dearborn, Michigan	Measuring perspectives of the meaning of diabetes self-management and its culture related barriers and facilitators amongst Arab American providers and patients	Five focus groups Two sessions involved n = 8 Arab American healthcare practitioners. Three sessions were conducted with n = 23 Arab American patients with T2D.	1- Individuals with diabetes have disclosed that their main obstacle to Diabetes Self-Management (DSM) is the absence of structured Diabetes Self-Management Education (DSME) programs. 2- Those suffering from Type 2 Diabetes (T2D) expressed that they do not experience stigma due to their condition. 3- Participants conveyed that DSM is challenging due to the stress they encounter, which they often attribute to familial circumstances.
Alzubaidi et al., 2016	Recruitment occurred at diabetes outpatient clinics in two tertiary referral hospitals, three primary care practices and two community centres.	To explore a new model for diabetes self-management support amongst Arabic-speaking immigrants in Australia.	Recruitment occurred at diabetes outpatient clinics in two tertiary referral hospitals, three primary care practices and two community centres. Total participants N = 60 N = 14 face-to face individual semi-structured interviews N = 8 group interviews involving 46 participants.	1- Participants conveyed that living with diabetes signifies a lifetime affliction and a significantly reduced quality of life. They indicated receiving information about self-management only upon diagnosis. This lack of comprehensive knowledge renders Arabic-speaking individuals less confident in their ability to control their diabetes. 2- Participants expressed feeling unsupported, stressed, and anxious. They expressed concerns that emotional distress related to diabetes is not sufficiently addressed during medical consultations. They appealed for increased emotional support and better education to handle daily stress levels. They also sought guidance on diabetes-friendly food options and appropriate daily portion sizes. Participants demonstrated a preference for in-person interaction and sharing of experiences over phone counselling or online-based interventions. Furthermore, they showed a preference for an Arabic-speaking physician or diabetes nurse educator to guide them in self-management, not only for language comprehension but also for cultural context understanding.

**Table 3:** Summary Characteristics of quantitative studies

First Author, Pub.Year.	Study Settings/ population/ duration/sample size	Study’s Aim	Study design/ tools used	Findings
Jansà,* et al., 2009	10 primary healthcare centers in Barcelona, Catalonia, Spain	To determine the profile of Moroccans with type 2 diabetes mellitus (T2DM), to provide diabetes education strategies.	cross-sectional study 70 questions covering: social demographic profile (11 questions); clinical data (16 questions); daily living with diabetes (attributions, knowledge about diabetes and quality-of-life, 25 questions); sociocultural factors 14 questions); appointment compliance and use of alternative medicine (4 questions)	• Interviews were conducted with forty patients, having an average age of 50 ± 15 years, with 73% being females. The predominant languages spoken were Arabic (58%) and Berber (42%). A majority of patients (90%) resided with their families, and 67% had spent more than five years in Catalonia. A significant percentage of patients (53%) were unable to read or write, 11% were on insulin therapy, 12% were administered oral medications, and 60% possessed capillary blood glucose meters. 1- It was reported by 66% of the patients that they had difficulties adhering to their dietary guidelines, while 44% found it challenging to maintain their medication schedules. 2- In terms of self-reported causes of Type 2 Diabetes Mellitus (T2DM), views on potential cures for diabetes, adherence to medication and appointments, and the use of alternative medicine: 60% of patients experienced language barriers. Furthermore, 63% of patients indicated that their impediments were related to observing Ramadan, and 86% participated in the Feast of the Lamb. 3- Among the patients, 11% were taking oral medications and 12% were on insulin, in contrast to the native population where these figures were higher, at 70.2% and 21.4% respectively. Additionally, 24% turned to alternative treatments, often herbal remedies.
Alzubaidi et al., 2015	Diabetes outpatient clinics at three major hospitals, ten general medical practices, and five community support groups.	To compare illness and treatment perceptions between ASB and ENG people with T2D. Also, exploring the decision-making processes and associated barriers and enablers that determine access and use of healthcare services in ASB and ENG patients with diabetes in Australia	A cross-sectional study used 12 validated tools measuring: diabetes-related distress, functional health literacy, treatment decision making, patients’ satisfaction with healthcare decisions, self-efficacy, and adherence to traditional values and attitudes (acculturation levels)	A high percentage of Arabic-speaking individuals (84%) exhibited low functional health literacy. Moreover, non-adherence to medication was seen in 88.3% of ASBs. A suboptimal level of blood sugar control and blood pressure was observed in 58.8% of ASBs, with HbA1c exceeding 7% (> 53 mmol/mol) and 13.85% having a blood pressure higher than 140/90mmHg. Additionally, Oral Hypoglycemic Agents (OHAs) were prescribed to 91.5% of the individuals. Compared to English-speaking participants, those who speak Arabic were significantly less compliant with all components of diabetes self-care, including dietary practices (P < 0.01; 95% confidence interval (CI) = − 1.17, − 0.84), physical activity (P = < 0.001, 95% CI = − 1.14, − 0.61), blood glucose testing (P < 0.001), and foot care (P < 0.001). Negative perceptions about diabetes among ASBs showed a strong and significant correlation with worse adherence to recommendations for diet, exercise, blood glucose testing, and foot care. Personal identity perceptions were significantly related to poor adherence to exercise, blood glucose testing, foot care, and dietary habits (Spearman’s rank correlation coefficients – 0.503; – 0.481– 0.228, – 0.494; respectively; P values < 0.001). 4- ASBs were less inclined to acknowledge the necessity for diabetes medications compared to English-speaking participants (P < 0.001, 95% CI = 0.93, 0.68). They also demonstrated heightened concerns regarding the usage and effects of diabetes medications (mean ± SD, 2.60 ± 0.86).
Alzubaidi et al., 2017	Diabetes outpatient clinics at three major hospitals, ten general medical practices, and five community support groups.	To examine and compare the patient–pharmacist relationship, medication underuse and adherence levels among Arabic speaking and Caucasian English-speaking patients with type 2 diabetes.	A cross-sectional study used 12 validated tools measuring: diabetes-related distress, functional health literacy, treatment decision making, patients’ satisfaction with healthcare decisions, self-efficacy, and adherence to traditional values and attitudes (acculturation levels)	Arabic-speaking individuals (ASBs) have been reported to have a lower compliance rate with pharmacist recommendations when compared to English-speaking individuals (ESBs), with 32% and 61.9% compliance rates respectively. ASBs were less prone to engage in conversations about their prescribed treatments with community pharmacists compared to ESBs, with the rates being 36% and 29% respectively. 3- Individuals holding a high school degree were almost twice as likely to seek a pharmacist’s advice concerning treatment or health issues, compared to those without a high school degree.
Berlie, et al., 2007	Cross sectional, population-based study conducted in Arab Americans during the years 2000 and 2001, In Dearborn, Michigan	To evaluate the quality of care among Arabic-speaking living with diabetes in United states. Also, comparing the results with other ethnic groups.	Cross sectional, population-based study conducted in Arab Americans during the years 2000 and 2001, assess the quality of care received by Arab American patients, Quality indicators (QI) such as the distribution of HbA1c, LDL, SBP and DBP were used to gauge the level of care received by the study patients. On the other hand, accountability measures were used to compare the quality of care in the Arab American population to that of the United States population	Over the past year, Arab Americans with diabetes reported visiting their physician once, with 75% of them stating they had four or more appointments. Merely 30% of the respondents achieved the American Diabetes Association (ADA) recommended HbA1c target of less than 7%. An average of 1.1 + 0.3 blood glucose tests per day and 4.6 + 3.8 tests per week were reported by 74% of participants. 66% of participants indicated that they had received diabetes education. Nutritional guidance was received by 93% of respondents, with 89% stating they adhered to the provided instructions. Strenuous exercise was partaken in by only 15% of Arab Americans, with a mere 5% engaging in non-strenuous physical activities. 70% of participants had an HbA1c of 7% or higher, and 25% had an HbA1c of 10% or higher. A greater proportion of female participants, 78%, had an HbA1c of 7% or higher, compared to 57% of males. A higher rate of non-completion of high school was noted among Arab Americans compared to other ethnic groups (41.5% NHANES vs. 27.1% BRFSS vs. 81.6% Arab American). Poor glycemic control, defined as HbA1c exceeding 9.5%, was comparable between Arab American females and the national female sample (21.9% vs. 20.3%), while Arab American males were more prone to poor glycemic control than the national male sample (33.3% vs. 16.1%). Arab American patients who hadn’t completed high school displayed higher rates of poor glycemic control compared to the national sample without a high school degree (30.0% vs. 16.1%). In the Arab American population, having diabetes for 15 years or more was associated with higher rates of poor glycemic control compared to the national sample (40.0% vs. 14.5%) . The likelihood of perceiving regular exercise as a crucial part of diabetes self-management was 70% lower for unemployed individuals compared to those who were employed [(Unemployed OR = 0.30, P = 0.046; 95% CI = 0.093–0.980)]. A significant relationship was observed between receiving instructions in Arabic and the outcome of food selection [OR = 6.66, P = 0.014; 95% CI = 1.48–30.03)]. A strong correlation was found between receiving instructions in Arabic and maintaining a healthy lifestyle [OR = 43.98, P = 0.003; 95% CI = 3.75-515.24)]. A considerable link was noted between family encouragement and engagement in discussions with clinicians regarding care [OR = 71.35, P = 0.003; 95% CI = 4.28-1189.06)]. There was a significant correlation between family understanding of food choices and participation in regular exercise [OR = 2.41, P = 0.03; 95% CI = 1.09–5.35)]. Participants voiced their concerns about dialogues with health-care providers and expressed a wish for doctors to provide more information and assistance regarding diabetes management. Participants also stressed the need to concentrate on the younger generations and educate them about a healthy lifestyle to foster a healthier community.
El Masri, 2020	To assess perceptions of diabetes self-management behaviors among ASBs with diabetes living in the US.	community pharmacies located in Michigan	For Quantitative analysis - Measure of central tendency were used to describe the sample - Pearson Chi-square tests were used to determine associations between perceptions of DSM behaviors and practices. - Logistic regression was also used to control for demographic factors and to obtain odds ratios between covariates and outcomes associated with perceived importance of DSM. behaviors. For Qualitative analysis: - data-driven inductive approach was used to establish codes and common themes.	Homemakers were 82% less likely to perceive regular exercise as an important DSM behavior. [(homemakers: OR = 0.187, P = 0.006; 95% CI = 056-0.620), The likelihood of perceiving regular exercise as a crucial part of diabetes self-management was 70% lower for unemployed individuals compared to those who were employed [(Unemployed OR = 0.30, P = 0.046; 95% CI = 0.093–0.980)]. A significant relationship was observed between receiving instructions in Arabic and the outcome of food selection [OR = 6.66, P = 0.014; 95% CI = 1.48–30.03)]. A strong correlation was found between receiving instructions in Arabic and maintaining a healthy lifestyle [OR = 43.98, P = 0.003; 95% CI = 3.75-515.24)]. A considerable link was noted between family encouragement and engagement in discussions with clinicians regarding care [OR = 71.35, P = 0.003; 95% CI = 4.28-1189.06)]. There was a significant correlation between family understanding of food choices and participation in regular exercise [OR = 2.41, P = 0.03; 95% CI = 1.09–5.35)]. Participants voiced their concerns about dialogues with health-care providers and expressed a wish for doctors to provide more information and assistance regarding diabetes management. Participants also stressed the need to concentrate on the younger generations and educate them about a healthy lifestyle to foster a healthier community.

### Quality Assessment

Two independent reviewers, AA and RK, conducted the assessment of quality for all the included studies, using two validated quality assessment tools based on the study design. For the quantitative studies, the Joanna Briggs Institute (JBI) Critical Appraisal tool was used to determine the bias of a study in its design, conduct and analysis [24]. The Hawker tool was used for the qualitative studies [25], it has 9-items and four levels of rating “good, fair, poor or very poor”. The associated tool allows us to convert these scales into a numerical score from 1 point for “very poor” to 4 points for “good”.

## Results

### Quality Assessment

The findings of quality assessment for both qualitative and quantitative studies are presented in Appendixes 1 and 2. Qualitative studies scoring varied between Good = 4, Fair = 3, Poor = 2, and Very Poor = 1 on the Hawker tool [25]. Overall, the five qualitative studies included in the review [19–21, 26, 27] were deemed good quality. The scores were 28/36 [27], 31/36 [21] and 34/36 [19, 20, 26].

In the five quantitative studies [28–32] the overall criteria were met using the JBI Critical Appraisal Checklist for Analytic Cross-sectional Study [19]. Of note, two studies conducted by Alzubaidi and his colleagues in 2015 and 2017 [30, 31] utilised the same sample. The research question, selecting target population, methods of recruitment and data analysis were deemed as appropriate in all studies. Sample size was calculated in four studies [28–32], while one study did not provide enough information regarding the sample calculation [28]. The details of statistical methods used were cited in all studies [28–32].

### Study Designs, Methodology, and Settings

Five quantitative studies utilising a cross-sectional design [28–32] and five qualitative studies [19–21, 26, 27] were included in the review. The methodology of qualitative studies varied, with two studies [21, 27] utilising only focus group interviews, while the other three [19, 20, 26] were conducted using both individual and focus group interviews.

Five studies were conducted in Australia [19, 20, 26, 30, 31], one in Spain [28] and four in the United States [21, 27, 29, 32].

### *Quantitative Study Participant Characteristics*

Two studies were conducted among first-generation Arabic-speaking immigrants [30, 31], two among those with Arab ancestry [29, 32] and one among immigrants from Morocco [28].

Overall, the sample size in these studies was 485. Participant characteristics are summarised in Table 2. There was a greater proportion of females compared to males across all studies (61% vs. 39%) [28–32]. All studies included participants 18 years and above, except one [29] with 25 years as the minimum age. The mean (SD) age of participants across all studies was (57.5 ± 4.2) years. The mean duration of living with diabetes (SD) was (7.7 ± 5.7) years. The education level among the participants across the five studies was grouped and categorized into three separate categories (did not attend; high school or less; and high school and above). Of the 485 participants, 10.1% did not attend school, 67.5% completed high school or less, and 20.5% completed high school and above [28–32]. Employment status was not grouped due to the variation in the presenting data between the studies. However, the results indicate that a large proportion of participants were not employed (66.4%). In addition, characteristics were not reported by gender. All participant characteristics are presented in Table 2.

### *Qualitative Study Participant Characteristics*

A total of 93 ASB participants were recruited across the five studies. Two studies did not mention the mean age [21, 27], while the mean age across the other three studies with the study population of first-generation Arabic immigrants [19, 20, 26] was 57 years. There were greater proportions of females compared to males across all the studies (60% vs. 40%). The average years of living with diabetes was nine years [19, 20, 26]. All participant characteristics are presented in Table 3.

### Self-management Strategies

Across all five qualitative studies, a number of self-management strategies were identified, such as attending regular appointments with health-care providers (HCP), adherence to prescribed diabetes medication, glucose monitoring, diet, and exercise [19–21, 26, 27]. Results are described according to the key strategies identified.

#### Monitoring of Disease Progression

Monitoring of disease progression includes attendance of regular medical appointments. Regular medical appointments is an indicator of patient engagement in diabetes care [33]. Three quantitative studies included in the review used a variety of terms for assessing this strategy: number of health-care provider visits [29]; if the participant attended the appointment [28]; and date of last health-care provider appointment [32]. The reported rate of attendance of medical appointments differed between the two studies that evaluated rate of attendance [28, 29] with health professionals. Berlie et al., found that all 53 ASB participants in the United States reported seeing their health-care providers at least once in the previous year, and 75.5% of them reported seeing their health-care providers four times or more in the same period [29]. However, Jansa et al., reported that 43% of ASB participants did not attend regular appointments with their health-care providers in Spain [28].

The health-care provider-patient relationship was discussed in all the included qualitative studies [19–21, 26, 27]. Participants stated that Arab-American health-care providers focussed mainly on medication prescription and overlooked patient-centred care – the approach that prioritizes the patient’s needs, wants, and preferences in medical decision-making and treatment [27]. Participants were reported to have negative perceptions of Arab health-care providers: they perceived that they received less care from Arabic-speaking health care providers compared to English-speaking health care providers [27]. According to Alzubaidi et al., consultations between Arabic-speaking primary healthcare providers and individuals from an Arabic-speaking background were short, with limited information provided about diabetes management. Many participants reported having uncertainties and unspoken concerns about the condition and its treatment. They expressed a desire for more information and a better discussion with healthcare providers [19]. However, a different study by Alzubaidi et al., found that ASB individuals with diabetes preferred Arabic-speaking healthcare providers because they shared the same language and cultural context [20].

#### Monitoring of Blood Glucose

Monitoring blood glucose, another diabetes management strategy, was reported in two quantitative studies [29, 30]. Berlie et al., reported that a majority [74%] of ASB participants tested blood glucose with participants, completing an average of 4.6 ± 3.8 tests per week [29]. In the same study, 30% of 53 participants met the level of HbA1c < 7, which follows American Diabetes Association (ADA) recommendations. It was also reported that more females adhered to this recommendation than males (78 and 57% respectively). Adherence to adequate glycaemic control of both sexes was also evaluated in the Alzubaidi et al., study, where the authors found that 58.8% of participants from both sexes had inadequate glycaemic control (HbA1c > 7) [30]. The study also found significantly (p < 0.001) lower levels of adherence [57.2%] to blood glucose monitoring among ASB participants as compared to those of English-speaking background (ESBs). In this present study, a patient is considered to be adhering to monitoring if they engage in blood glucose monitoring for more than 3 of the previous 7 days [30].

#### Adherence to Anti-diabetic Medications

In addition to making regular meetings with health-care providers, adherence to prescribed medication is a significant part of diabetes management [33]. All the included quantitative studies [28–32] reported on adherence. There was variation in the methods of reporting information on adherence, including questions such as the number of participants who ceased taking medications without medical advice [28], and use of validated questionnaires such as the Morisky scale [30, 31]. The use of oral glucose-lowering agents ranged from 12 [21] to 91.5% [30, 31]. One study [29] collated information on medication utilization but not on adherence. Jansa et al., reported that of 40 participants 32% stopped taking their medication without medical advice [28]. In the same study [28], use of alternative medicines, mainly herbal, was reported among 24% of participants. Two studies with the same study population found that 63.5% of participants from ASB had difficulties following their medication regimes [30, 31]. Alzubaidi et al., who examined and compared medication use, level of adherence and the relationship between pharmacists and people living with T2DM from ASB and English-speaking background (ESBs), found that only a minority from ASB individuals were following pharmacist recommendations compared with their counterparts from ESB (32% vs. 61.9%, respectively); in addition, they were also less likely to discuss their prescribed treatment with community pharmacists compared to participants from ESB (36% vs. 29%, respectively) [31].

#### Diet

Apart from medical management, other self-management strategies also include adherence to healthy diet, which was assessed in four quantitative studies [28–30, 32]. Difficulties in following diabetic-specific diets among the studied population varied from 66% [28] to 89% [29]. Alzubaidi et al., reported that participants from ASB had a lower rate of adherence to dietary behaviours compared to participants from ESB (95% CI = − 1.17 to − 0.84) [30]. The study by El-Masari et al. evaluated the association between having instructions delivered in the Arabic language and food selection and found a significant association between the two factors (OR = 6.66, p = 0.01) [32].

One qualitative study [20] also found that participants reported a lack of dietary knowledge. Participants’ discussion on traditional diets and culture, and the ways these can facilitate or hinder DSM education, were discussed in another study [20]. For an example, Ramadan, an important cultural event, could result in superseding the advice of providers and impede effective DSM education [21]. Another reason highlighted was the associated stigma: participants mentioned that they were not likely to disclose their condition, especially in a social gatherings, due to fear of causing offense or disrespect to the host by refusing the offered food [26].

#### Adherence to Physical Activity

Other self-care activities are also important cornerstones for diabetes care. Alzubaidi et al., reported that participants from ASB had low levels of adherence to self-care activities compared to participants from ESB, including in physical activity (95% CI = − 1.14, − 0.61) [30]. Likewise, Berlie et al., found that only 15% of participants from ASB engaged in strenuous exercise and 5% engaged in non-strenuous physical activity [29].

In the context of physical activity, culture and its possible impact were discussed in one qualitative study [21]: it was, for example, considered inappropriate for females to participate in mixed-gender exercise programs.

#### Factors Impacting Self-management Strategies

Beliefs also play a crucial role in DSM. This includes beliefs and perceptions towards the necessity of taking diabetes medications. Alzubaidi et al., reported that participants from ASB were less likely to see the need for diabetes medications compared with participants from ESB (10.7% (ASB) vs. 51.7% (ESB); 95% CI = (0.93, 0.68) [30]. In addition, based on the average of Beliefs about Medicines Questionnaire (BMQ), that was conducted by Alzubaidi et al., participants were assigned to one of the four groups (accepting, ambivalent, indifferent or sceptical), and individuals from ASBs showed greater concern about the medication’s side-effects compared to those from ESB (ASBs mean ± SD, 2.60 ± ESBs mean ± SD, 0.86, 3.41 ± 0.76, respectively) [30]. Likewise, more than half of participants from ASBs were more sceptical compared to participants from ESBs (52.8% vs. 11.2%), and they showed less acceptance of diabetes treatment compared to the same group (14.4% vs. 51.3%) [30]. Alzubaidi et al., showed that people who had positive perceptions towards the ability to control their diabetes were more likely to engage in recommended behaviours, including exercise, blood glucose testing and foot care (Spearman’s rank correlation coefficients 0.28, 0.27, 0.24, respectively; p < 0.001) [30].

Some of the reasons and beliefs contributing to lower adherence to diabetes medication among people from ASB were reported in two qualitative studies [19–26]. Participants from ASB had difficulties of adherence due to their fear of long-term side-effects and lack of trust in Western medicine [19]. It was also suggested that individuals from ASB had a negative social view of diabetes and associated taking prescriptive medications for diabetes as a sign of sickness and fragility [26]. In addition, people from ASB commented on the significance of their social image or peers’ views: they believed that being a person living with diabetes or taking diabetes medications equated to shame or stigma, thus leading them to hide their condition and not take medications, especially in social gatherings [26]. Another qualitative study [19] reported religious beliefs that may play a role in the management of diabetes among some participants: it reported people’s fatalistic attitudes, suggesting that human life is not eternal, it is only transient, and it was argued that people should not engage in healthy lifestyle activities since eventually they will die in any case. They also believed that suffering from illnesses such as diabetes would make them closer to Allah (God) [19].

It was suggested that the education level of individuals from ASB also had an impact on diabetes management [29, 30]. For instance, Alzubaidi et al., found that people with a high school qualification were almost twice as likely than those who did not have a high school qualification to consult a pharmacist when they had issues with health or medications (P < 0.008) [30]. Similarly, immigrants from ASB in the US who did not complete high school had poorer glycaemic control compared to non-ASB immigrants of similar educational levels (30.0% vs. 16.1%) [29].

Lack of employment also contributed to poor DSM. El Masri et al., concluded that those who were homemakers (82%) or unemployed (70%) were less likely to perceive regular exercise as an important DSM behaviour compared with those who were employed (homemakers: OR = 0.18, 95% CI = 0.56–0.62; unemployed: OR = 0.30, 95% CI = 0.093–0.98) [32]. These findings were in contrast to the study by Alzubaidi et al., which found that participants who were working a part-time job were found to be less likely to follow recommendations by 3.5 times (95% CI 1.2 to 10.2) compared to those who were full-time workers [31].

An additional factor that impacted DSM was that language barrier, which was noted amongst 60% of participants [28]. El Masri et al., noted that there was a significant association between receiving instructions in Arabic and outcome of food selection (OR = 6.66, 95% CI = 1.48–30.03) and maintaining a healthy lifestyle (OR = 43.98, 95% CI = 3.75–515.2) [32].

Lack of understanding of self-management strategies was the one of the most reported reasons, mentioned in three qualitative studies [19, 20, 27]. Alzubaidi et al., found that individuals from ASB reported having received most of the DSM information (oral and written) at the time of diagnosis but it was poorly understood and may be forgotten over time [19]. The lack of understanding related to the seriousness of diabetes, diabetes treatment, diabetes-related health services, diabetes-friendly food, amount of physical exercise, and the portion of food that may be appropriate for people living with diabetes [20]. The same authors in another study concluded that, with the lack of culturally adapted DSM education programs, patients felt ill-equipped to adopt self-management strategies [26]. As a result of lack of continuous and culturally inappropriate diabetes information, ASB participants were reported to have knowledge deficits regarding DSM strategies as well as non-medical services provided for diabetes management [19, 21].

Another factor impacting the adherence level to DSM was the high level of diabetes-related stress among people from ASBs living with diabetes [19, 27]. Alzubaidi et al., reported that immigrants from ASB living with diabetes felt powerless, anxious, and overwhelmed in dealing with diabetes [19]. The level of stress can impact on the likelihood of people adopting self-management strategies in the management of T2DM. Firtz et al., reported that participants from ASB expressed that daily life stress affected their ability to control diabetes [27].

## Discussion

The study found low adherence to diabetes self-management (DSM) strategies, including disease progression monitoring, blood glucose monitoring, taking diabetes medication, following a diet, and engaging in physical activity, among individuals from ASB communities. The review identified various factors that impact DSM among ASB immigrants, such as beliefs, education level, unemployment, language barriers, lack of understanding of diabetes self-management, and social influence and diabetes-related stress. It is noteworthy that this review included studies across multiple Western countries. Yet it appears that the reported barriers to DSM are similar across different settings, indicating that they are universal for ASB populations.

In comparing our findings with those from studies [34–42] conducted on other ethnic groups, it becomes evident that cultural context plays a pivotal role in diabetes self-management across diverse populations. Culture has been shown to have a substantial impact on the adherence to diabetes self-management strategies. Cultural beliefs, values, and practices can shape an individual’s understanding, perception, and management of their health and illness, including diabetes. Research has demonstrated that cultural factors, including traditional beliefs, dietary habits, and familial support, can affect the adoption and persistence of self-management behaviours such as diet regulation, physical activity, and medication utilization. For instance, a study conducted by Brown et al., determined that cultural beliefs concerning the causes and treatments of diabetes, as well as conventional dietary practices, constituted significant impediments to diabetes self-management for African Americans with Type 2 diabetes [34]. Similarly, findings among Hispanic individuals revealed that prayer is often used as a form of spiritual treatment for diabetes, which can consequently lead to delays or neglect in seeking appropriate medical treatment [42]. Furthermore, Li et al., found that cultural values and beliefs, including individualism, fatalism, and collectivism, have a significant influence on the level of self-care and health-seeking behaviours among individuals with diabetes [35].

Effective communication between patients and healthcare providers can raise the patient’s understanding and knowledge, as well as foster a collaborative atmosphere rooted in mutual trust. This can prompt patients to openly address their problems and concerns and has significant implications for the development of a culturally and religiously sensitive diabetes education approach for ASB immigrants. New educational approaches that take into account cultural and religious aspects, such as incorporating storytelling, visual and written resources in the native language, and offering activities like healthy food purchasing and preparation, supermarket tours, and cooking classes, may aid in promoting a healthy lifestyle for immigrants [36]. The present review supports the necessity to encourage health-care providers to be more proactive and discuss the best strategies to control diabetes with their patients from ASB and encourage them to utilise interpreting services. Furthermore, health coaching, which is a “patient-centred, patient-decided approach to disease management” [37], may be a useful tool to apply when controlling chronic disease, especially diabetes. Some studies showed that health coaches can help people with chronic diseases to engage more in self-management and improve their overall health [38]. Individuals from ASB may benefit from engaging in health coaching to improve their self-management activities. Such an approach has been reported to have positive returns on investment and it may decrease morbidity rate from diabetes and the costs associated with normal medical care [39].

One of the challenges in evaluating adherence to self-management strategies in quantitative studies is the lack of commonly used outcome measures. This makes it difficult to compare results across different studies and to determine the effectiveness of various self-management strategies. In addition, the cultural appropriateness of these outcome measures is also a concern. Culture can have a significant impact on the attitudes, beliefs, and behaviours related to self-management [40], and culturally insensitive or inappropriate outcome measures may not accurately capture the experiences and perspectives of individuals from different cultural backgrounds [41]. It is important for researchers to critically evaluate the cultural sensitivity and appropriateness of outcome measures used in self-management studies, and to consider the development of culturally sensitive and inclusive outcome measures that are relevant and meaningful for diverse populations. This will help to ensure that self-management research is culturally responsive and that the results are applicable to a wide range of individuals, including those from diverse cultural backgrounds.

## Strengths and limitations

To the best of our knowledge, this is the first systematic review identifying barriers and enablers of DSM strategies among ASB immigrants living with type 2 diabetes in high-income Western countries. Qualitative and quantitative studies that have been included in this review provide a comprehensive analysis of studies in this area. One of the limitations in this review was the inability to conduct a meta-analysis, due to the lack of homogeneity of outcome measures. A majority of the studies reviewed recruited participants from hospitals, clinics, and/or community centres, which may not represent the whole community, and this will impact the generalizability of the study. Another limitation was that of the ten studies included, five were performed by one author group [19, 20, 26, 30, 31] and conducted in one country (Australia). The limited sample size and geographic focus on Australia may not fully capture the diversity and experiences of the global Arabic-speaking immigrant population, thereby affecting the generalizability of our findings. These limitations support the need for further research targeting this ethnic group that includes a more diverse and larger sample size that spans multiple geographic locations to enhance the validity and applicability of the study.

This study acknowledges the value that could be added through a comparison between individuals from Arabic-speaking backgrounds (ASB) and those from other ethnic groups. The researchers recommend that future studies adopt a more comprehensive approach by including this comparative element. Although the scope of the current study precluded such a detailed comparison, there is significant merit in future research efforts that address this limitation to yield a more comprehensive understanding of diabetes self-management across diverse cultural and ethnic contexts.

## Conclusion

This systematic review summarizes relevant studies regarding barriers and enablers of DSM among Arabic-speaking background living with type 2 diabetes in high-income Western countries. Despite living in these high-income countries, ASB immigrants face challenges to adoption of self-management strategies. These challenges include religious beliefs, lack of knowledge about self-management and language barriers. The current review suggests that there is a need for a diabetes self-management educational program (DSME) that is tailored to ASBs language, culture and beliefs. It also points out that health-care providers, in cooperation with religious leaders, play a significant role in improving health outcomes among this ethnic group.
